# Topology and adenocarcinoma cell localization dataset on the labyrinthin diapeutic biomarker

**DOI:** 10.1186/s13104-023-06373-4

**Published:** 2023-07-06

**Authors:** Ankit Sharma, Michael Babich, Tianhong Li, James A. Radosevich

**Affiliations:** 1 LabyRx Immunologic Therapeutics Limited, 2700 Stockton Blvd, Sacramento, CA 95817 USA; 2 Division of Hematology & Oncology, University of California Davis Comprehensive Cancer Center, Sacramento, CA USA

**Keywords:** Pan-tumor target, Biomarker, Neo-antigen, Adenocarcinoma, Tumor associated antigen, Tumor specific antigen, Labyrinthin, ASPH

## Abstract

**Objective:**

The discovery and characterization of tumor associated antigens is increasingly important to advance the field of immuno-oncology. In this regard, labyrinthin has been implicated as a neoantigen found on the cell surface of adenocarcinomas. Data on the (1) topology, (2) amino acid (a.a.) homology analyses and (3) cell surface localization of labyrinthin by fluorescent activated cell sorter (FACS) are studied in support of labyrinthin as a novel, pan-adenocarcinoma marker.

**Results:**

Bioinformatics analyses predict labyrinthin as a type II protein with calcium binding domain(s), N-myristoylation sites, and kinase II phosphorylation sites. Sequence homologies for labyrinthin (255 a.a.) were found vs. the intracellular aspartyl/asparaginyl beta-hydroxylase (ASPH; 758 a.a.) and the ASPH-gene related protein junctate (299 a.a.), which are both type II proteins. Labyrinthin was detected by FACS on only non-permeablized A549 human lung adenocarcinoma cells, but not on normal WI-38 human lung fibroblasts nor primary cultures of normal human glandular-related cells. Microscopic images of immunofluorescent labelled MCA 44-3A6 binding to A549 cells at random cell cycle stages complement the FACS results by further showing that labyrinthin persisted on the cell surfaces along with some cell internalization for greater than 20 min.

## Introduction

The identification and characterization of neo-antigens [1–3] are rare yet increasingly important for cancer diagnostics and therapeutics. Labyrinthin is a neo-antigen example in that it is selectively expressed on the cell surface of adenocarcinomas but is not found on normal or other cancer cell types [3, 4]. This is significant because adenocarcinomas represent about 40% of all solid cancer types and over70% of cancer-related deaths [4]. The present data extends and supplements a recent report [4] on labyrinthin as a distinguishing marker for adenocarcinomas vs. other targets. The results indicate that therapeutic strategies can be employed that take advantage of labyrinthin’s strategic presence on adenocarcinoma cell surfaces (e.g., antibody accessibility). Furthermore, the discovery of a calcium binding sequence in labyrinthin provides a basis to research if it is key to explaining the long-known phenomena of elevated calcium and irregular signaling in cancer cells [5, 6]. The data also reveal a need to explore distinctions between ASPH and labyrinthin, in particular because ASPH [7, 8] and labyrinthin-based treatments have already been FDA-approved for clinical trials.

## Materials and methods

### Labyrinthin primary structure analysis: Topology and sequence homologies

The amino acid sequence for labyrinthin obtained from the National Center for Biotechnology Information was used for general topological analysis and for alignment and antibody epitope comparisons with junctate and ASPH.

### Topology of labyrinthin

The sequence was entered into various programs for analysis by several computer programs, each with distinct advantages and areas of focus (e.g., MolBio-Tools.ca). The online tools used to analyse various topology characteristics include Isoelectric.org, Prot pI, Pepcalc, ScanProsite, TMPred, Quick2D, Protter, PROTEUS2, MemBrain, TMHMM v2.0.

### Sequence comparisons

BLAST analysis (Basic Local Alignment Search Tool) was conducted to determine sequence similarities with labyrinthin. The two significant homologies, junctate and ASPH, were then aligned to display the similarities. The sequences were then scanned for known epitopes for commonly used antibodies against labyrinthin and ASPH or junctate (MCA 44-3A6 and FB-50, respectively).

### Fluorescent activated cell sorter (FACS) analysis

FACS was performed independently (Stanford Research Institute International; California, USA) as follows:

### Reagents and cell lines

Mouse monoclonal anti-labyrinthin antibody MCA 44-3A6 was provided by the current lab [9] as grown using standard hybridoma technology and purified by 50% saturated ammonium sulphate precipitation and ion exchange chromatography or by a commercial kit (Pierce™ Antibody Clean-up Kit). Cytofix/Cytoperm solution, Perm/Wash buffer, biotinylated goat anti-mouse antibody, Fluorescein-5-isothiocyanate (FITC)-conjugated goat anti-mouse antibody, FITC-conjugated streptavidin, and propidium iodide were obtained from Becton Dickinson. BSA, trypan blue, and sodium azide were obtained from Sigma, and PBS (phosphate buffer saline) was purchased from Media Tech. Cell lines were obtained and grown as recommended by the American Type Culture Collection (Manassas, VA). Labyrinthin-negative WI-38 normal human lung fibroblast cells (negative control) and A549 human lung adenocarcinoma (positive control) cells were used in this experiment. Because adenocarcinomas are neoplasia of epithelial tissue that have a glandular origin, normal primary cultures of human astrocytes, renal proximal tubule epithelial cells, and small airway epithelial cells were selected as counter models in this study.

### Cell staining and Flow Cytometry

Cells were counted using a hemocytometer and stained with trypan blue to visualize dead cells and cells were stained at about 500,000 cells/tube. For extracellular staining, cells were incubated with the indicated concentrations/dilutions of 44-A36 antibody for 30 min on wet ice and then washed twice with PBS containing 1% BSA and 0.05% sodium azide (FACS buffer). Cells were then incubated with indicated concentrations of biotinylated goat anti-mouse antibody for 30 min on wet ice (in some experiments, biotinylated goat antibody was diluted in normal goat serum). After washing twice with FACS buffer, cells were incubated with the indicated concentrations of FITC-conjugated streptavidin for 30 min on wet ice, followed by washing twice with FACS buffer. Propidium iodide was added to cells immediately before FACS analysis. Unstained controls were not incubated with 44-A36 antibody, but were incubated with all subsequent antibodies. FACS cell labeling confirmation and detection of labyrinthin at various cell cycle stages.

Cells used in previous FACS studies [Fig. 2] were examined to (1) confirm sufficient labeling and (2) observe labyrinthin localization at random stages of the cell cycle. A sample of A549 cells grown to 50–80% confluence and prepared for FACS analysis were viewed under a glass coverslip by fluorescence microscopy. Images were obtained 20–30 min following antibody incubation and cell washing. Importantly, all of the cells types were scraped off of the tissue culture flasks and trypsin/EDTA treatment was NOT used for any of the cell harvesting protocols.  

## Results

### Labyrinthin topology and amino acid sequence comparisons with junctate and ASPH

**Fig. 1:**
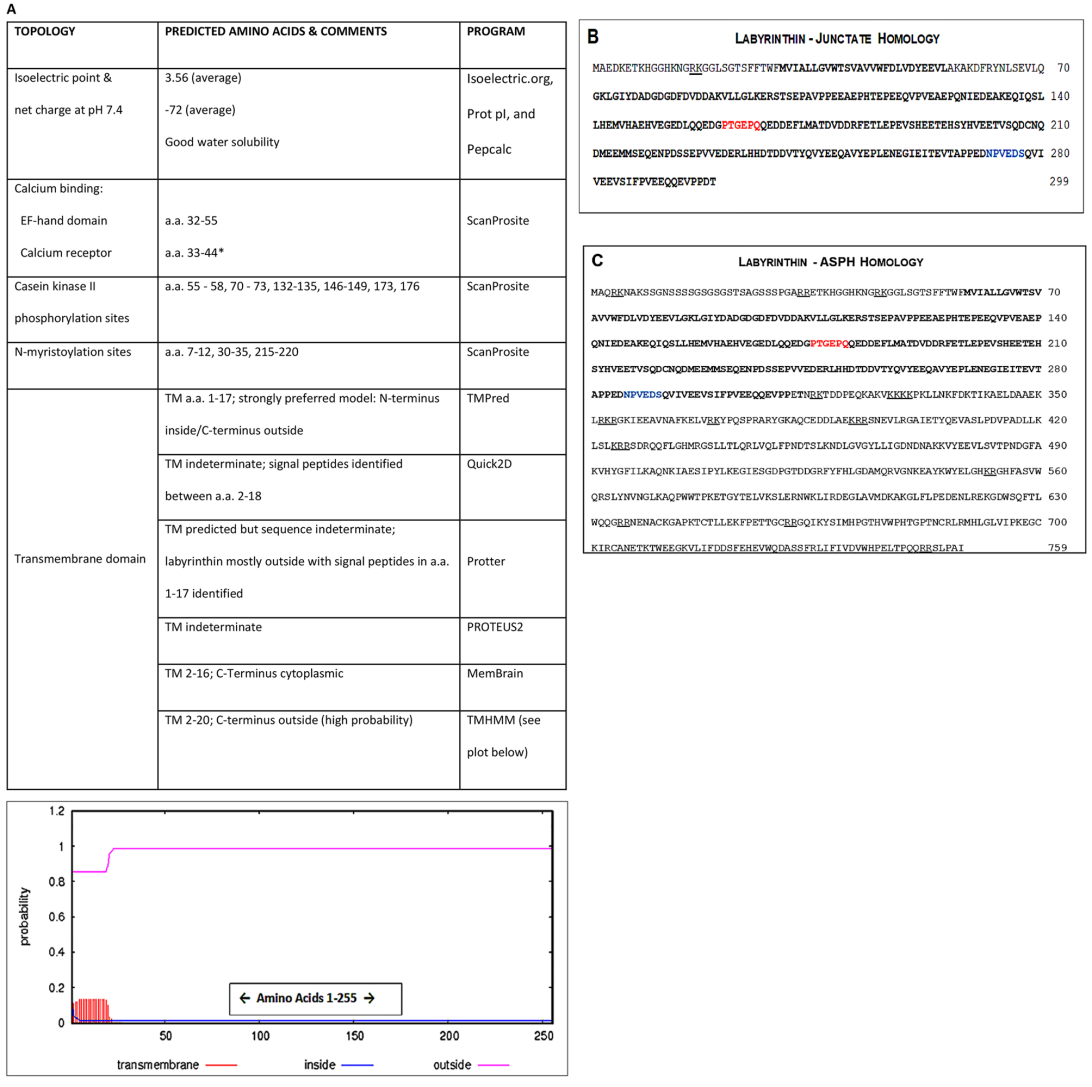
Labyrinthin topology and amino acid sequence comparisons with junctate and ASPH. (**A**) Labyrinthin topology as analyzed by the given computer programs. The TMHMM plot is shown because it most closely depicts a composite of the results. (**B**) Junctate and (**C**) ASPH (a.k.a., AAH, HAAH) are compared to the primary structure of labyrinthin (**bold** lettering). Epitopes for mouse monoclonal anti-labyrinthin antibody MCA 44-3A6 and the widely used FB-50 anti-ASPH antibody are shown in red and blue, respectively. RK/KR and KKXX motifs essential for ER targeting of proteins, and RR motifs that maintain type II proteins in the ER are underlined (bars). *Partial sequence identified

Labyrinthin has been implicated as a pan-tumor marker and target [3, 4], but more information is needed about elementary attributes related to its structure. Therefore, various computer-based programs were used to evaluate labyrinthin topology (Fig. 1A). The analyses shows that labyrinthin is a very acidic protein with standout features that include: (1) a partial calcium binding domain and (2) casein kinase II phosphorylation sites that are considered important for protein function or signalling mechanism(s); (3) a signal peptide for labyrinthin that coincides with N-myristoylation [10] that is consistent with N-terminus membrane localization as a type II protein [11]; and (4) a transmembrane domain (though indeterminate by some programs).

The amino acid sequence of labyrinthin (as per Gene Bank) was used to determine homologies with other human proteins by the Basic Local Alignment Search Tool (National Center for Biotechnology Information). Four major proteins were identified that are generally considered intracellular: ASPH, and ASPH gene-related junctate, junctin and humbug. The entire sequence of labyrinthin can be found within junctate (Fig. 1B), but it is missing the endoplasmic reticulum (ER)-targeting sequence (junctate a.a. 56–70). The humbug sequence (313 a.a.) contains junctate and therefore has homology with labyrinthin; it is 99.68% homologous with the N-terminus of ASPH that represents 41% of complete humbug-ASPH sequence comparison. Other than a substitution at ASPH position 312 (glutamic acid instead of aspartic acid), there are no sequences in labyrinthin that are not contained within ASPH (Fig. 1C). Only 50 amino acids in junctin (225 a.a.) share identity with labyrinthin (a.a.#29–95; not shown).

Epitopes for both mouse monoclonal anti-labyrinthin antibody MCA 44-3A6 [12] and the widely used FB-50 anti-ASPH antibody [7, 8] were identified in labyrinthin, junctate, and ASPH (Fig. 1B C); junctin (not shown) lacks the epitopes for both antibodies. Though antibody cross-reactivity is likely, localization of labyrinthin (cell surface) would help distinguish antibody signal vs. intracellular junctate and ASPH. Indeed, the essential ER-targeting arginine/lysine-rich motifs (RK/KR) and lysine (K) with any amino acid (X) motifs (e.g., KKXX), and RR motifs that maintain type II proteins in the endoplasmic reticulum (ER) [13–15], are only present in ASPH and junctate (Fig. 1B C).

### Selectivity of cell surface adenocarcinoma marker labyrinthin by FACS analysis

**Fig. 2:**
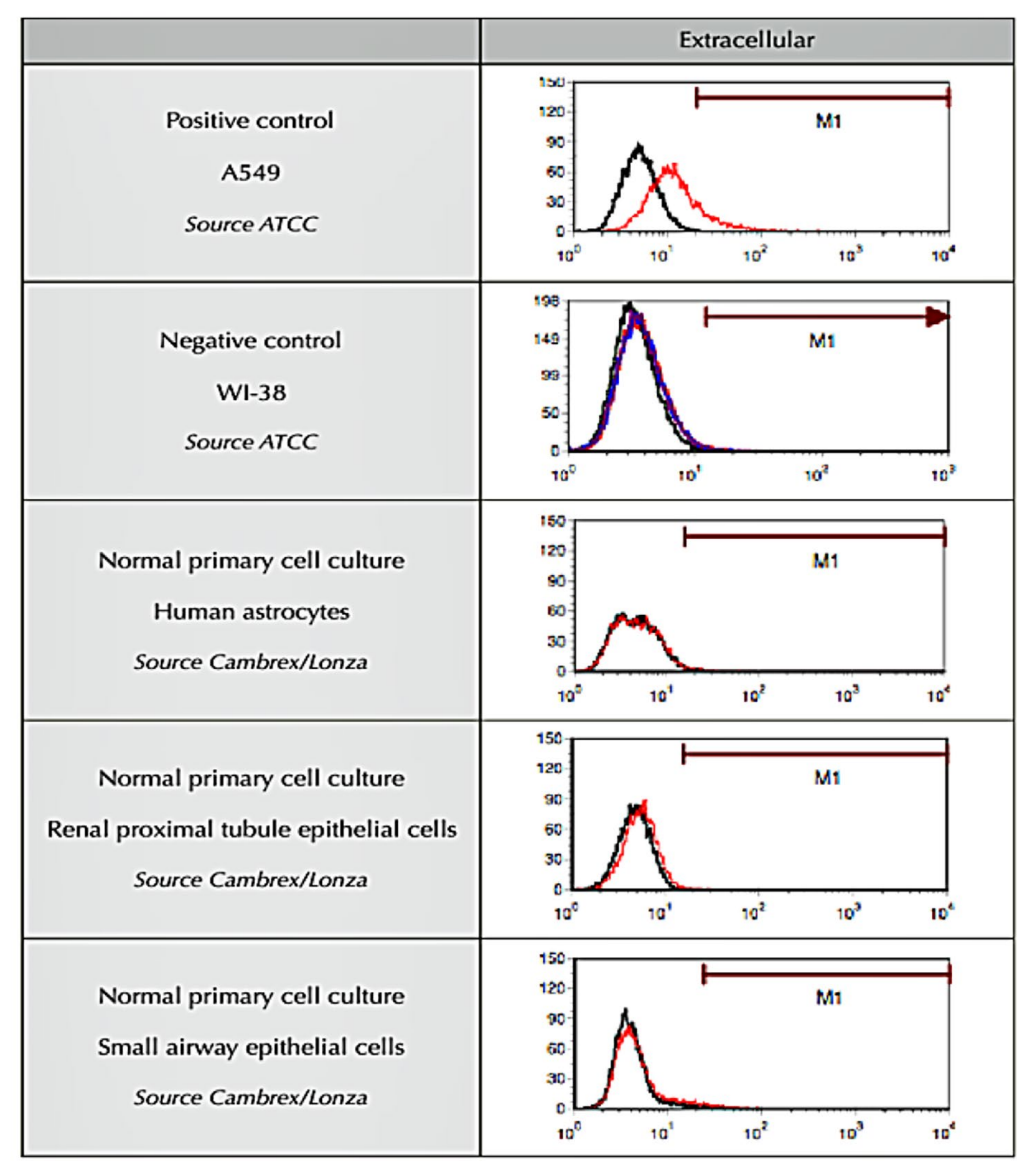
Selectivity of cell surface adenocarcinoma marker labyrinthin by FACS analysis.The presence or absence of labyrinthin on non-permeabilized human A549 lung adenocarcinoma and normal cells is shown. Experiments were performed by a third-party (Stanford Research Institute International, Menlo Park, CA). Tracings represent results from at least duplicate preparations for each of the cell cultures

The absence of labyrinthin on normal cells, and the specific localization to the cancer cell surface, is essential for it to be a viable neo-antigen marker and target. The lack of labyrinthin on normal cells/tissues could minimize side effects of potential therapeutics. Such characteristics also serve to distinguish it from other putative pan-cancer targets such as ASPH. Independent third-party confirmation of labyrinthin on using human A549 lung adenocarcinoma cells as compared to normal cells was studied by FACS analysis (Fig. 2). A549 and WI-38 normal lung fibroblasts were respectively shown to be positive and negative control cells for labyrinthin, as expected. To support the idea that the tumor marker is not associated with normal tissues and, as such, is a neo-antigen, labyrinthin was not detected on primary cultures of normal human astrocytes, renal proximal tubule epithelial cells, and small airway epithelial cells. It should be noted that in preliminary work, the following non-adenocarcinoma cancer cells were also negative for labyrinthin: BT 20 mammary gland carcinoma, T47D mammary gland ductal carcinoma, and SSC15 squamous cell lung carcinoma. In addition, MCF7 mammary adenocarcinoma and SCC40 tongue squamous cell carcinoma cells were anomalies that were negative and positive (8.8%), respectively.

### Immunofluorescence of MCA 44-3A6 binding to intact A549 cells: cell cycle and surface localization

**Fig. 3:**
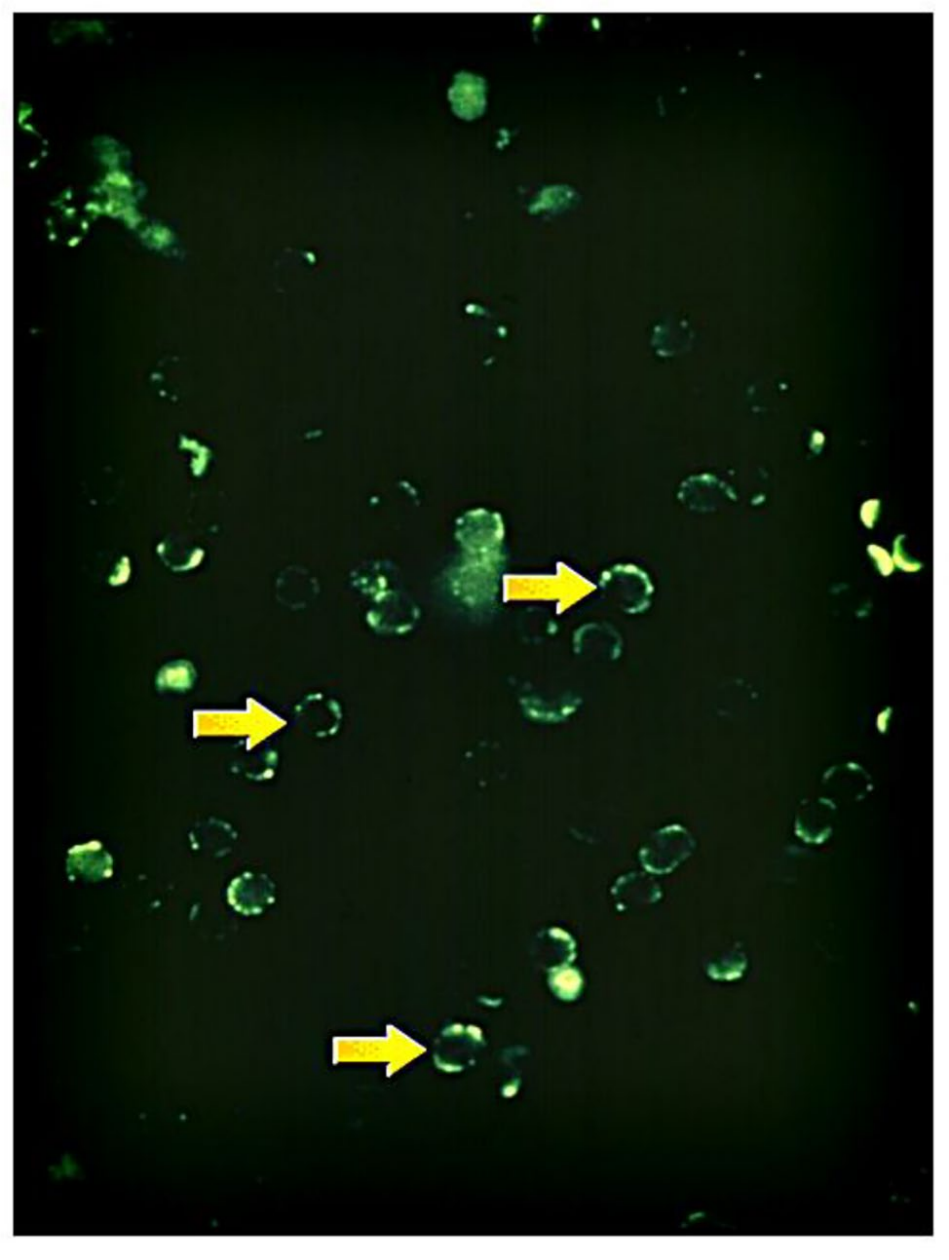
Immunofluorescence of MCA 44-3A6 binding to intact A549 Cells: Cell cycle and surface localization. A549 cells at random stages of their cell cycle were incubated with immunofluorescent labelled MCA 44-3A6 antibody and examined within 20–30 min by fluorescence microscopy (100x shown). Labyrinthin localization is shown (arrows)

A portion of the cells labelled by MCA 44-3A6 used for FACS experiments was set aside to check for sufficient labelling and to assess post-labeling localization of labyrinthin. The cells are not permeabilized so the results visualize antibody location after initial exposure and targeting cell surface epitopes. Intense staining is noted on cell surfaces (Fig. 3) with some internalization of antibody and/or antibody-labyrinthin. Positive intracellular signal was also seen that is consistent with labyrinthin internalization, cycling, and/or ER trafficking [9].

## Discussion

Identification of potential markers that can also serve as therapeutic targets are presently an important trend in cancer research. The present work elaborates on labyrinthin as a tumor specific antigen and potential diapeutic (diagnostic + therapeutic) target for adenocarcinoma cancer types. Indeed, labyrinthin is at present being used to screen adenocarcinoma patients for treatment directed against this antigen (clinicaltrials.gov).

Labyrinthin has sequence homology with members of the ASPH gene family but is distinct with respect to having a role as both a cancer marker and target [4]. The present work extends the previous studies to show that labyrinthin is specifically expressed on adenocarcinoma cells and is available throughout the cell cycle. The results also raise the question of whether there is a physiological signalling role for labyrinthin after internalization, such as occurs with G-protein coupled receptors [16]. A possible regulatory role of labyrinthin in intracellular signalling may be implicated due to the presence of a partial calcium binding domain. In this regard, (1) elevated and unregulated levels of the second-messenger calcium has long been observed in cancer; (2) the possible role of type II proteins in intracellular calcium homeostasis via calsequestrin binding was recently suggested [4] and, (3) labyrinthin expression was increased in A549 cells exposed to elevated calcium [17], indicating some interplay between calcium and labyrinthin.

In summary, a diapeutic role for labyrinthin is supported by the specific association and convenient localization on the surface of adenocarcinoma cells, whereas the absence of labyrinthin on the surface of normal cells can ensure minimizing the side effect of labyrinthin-targeted therapy.

### Limitations

- This study doesn’t show the possible physiological role of internalized labyrinthin.
- Computer modeling does not necessarily reflect real-world dynamics of labyrinthin topology.

## Data Availability

With the article and “Topology and adenocarcinoma cell localization dataset on the labyrinthin biomarker”, Mendeley Data DOI:10.17632/pg7z5zkgc8.1. https://data.mendeley.com/datasets/pg7z5zkgc8/1. NCBI GenBank Accession Number (labyrinthin): AFL00846. https://www.ncbi.nlm.nih.gov/protein/AFL00846.1. “Amino acid sequence homology with labyrinthin”, RID-3T92WK3S013. https://blast.ncbi.nlm.nih.gov/Blast.cgi?CMD=Get&RID=3TAAG80A013. NCBI GenBank Accession Number (ASPH): NP_004309.2 https://www.ncbi.nlm.nih.gov/protein/NP_004309.2. NCBI GenBank Accession Number (junctate): AAG42257. https://www.ncbi.nlm.nih.gov/protein/AAG42257. NCBI GenBank Accession Number (junctin): AAF82247. https://www.ncbi.nlm.nih.gov/protein/AAF82247. NCBI GenBank Accession Number (humbug): NP_115855.1. https://www.ncbi.nlm.nih.gov/protein/NP_115855.1.

## Abbreviations

FACS: fluorescent activated cell sorter
ASPH: aspartyl/asparaginyl beta-hydroxylase
FITC: Fluorescein-5-isothiocyanate
PBS: phosphate buffer saline
ER: endoplasmic reticulum

## References

[1] Zhang Z, Lu M, Qin Y, Gao W, Tao L, Su W, Zhong J (2021). Neoantigen: a new breakthrough in Tumor Immunotherapy. Front Immunol.

[2] Ma W, Pham B, Li T (2021). Cancer neoantigens as potential targets for immunotherapy.

[3] Radosevich JA, Babich M. 2019. Labyrinthin, the tumor marker recognized by MCA 44-3A6: a case for pan-tumor markers as targets to treat Cancer. OncoTargets and therapy, 12, p.9351.PMID: 31807015. 10.2147/OTT.S220445 PMC6844151 31807015

[4] Babich M, Sharma A, Li T, Radosevich JA. 2022. Labyrinthin: A distinct pan-adenocarcinoma diagnostic and immunotherapeutic tumor specific antigen. Heliyon, p.e08988., PMID: 35252607. 10.1016/j.heliyon.2022.e08988 PMC8891966 35252607

[5] Banyard MR, Tellam RL (1985). The free cytoplasmic calcium concentration of tumorigenic and non-tumorigenic human somatic cell hybrids. Br J Cancer.

[6] Stewart TA, Yapa KT, Monteith GR (2015). Altered calcium signaling in cancer cells. Biochim et Biophys Acta (BBA)-Biomembranes.

[7] Kanwal M, Smahel M, Olsen M, Smahelova J, Tachezy R (2020). Aspartate β-hydroxylase as a target for cancer therapy. J Experimental Clin Cancer Res.

[8] Wands JR, De La Suzanne M, Deutch AH. HA Ghanbari 2001. Diagnosis and treatment of malignant neoplasms. U S Patent 6,835,370, Patent ID.

[9] Radosevich JA, Siddiqui FS, Rosen ST, Kabat WJ. 1991. Cell cycle and electron microscopic evaluation of the adenocarcinoma antigen recognized by the monoclonal antibody 44-3A6. The British journal of cancer. Supplement, 14, p.86. PMID: 2039713. PMC2204118 2039713

[10] McIlhinney RJ (1998). Membrane targeting via protein N-myristoylation. Protein targeting protocols.

[11] Hartmann E, Rapoport TA, Lodish HF. 1989. Predicting the orientation of eukaryotic membrane-spanning proteins. Proceedings of the National Academy of Sciences, 86(15), pp.5786–5790.PMID: 2762295. 10.1073/pnas.86.15.5786 PMC297715 2762295

[12] Radosevich JA, ImmvaRx, Inc. 2000. Cancer marker protein and peptides thereof. U S Patent 6,166,176, Patent ID.

[13] Schutze MP, Peterson PA, Jackson MR. 1994. An N-terminal double‐arginine motif maintains type II membrane proteins in the endoplasmic reticulum. The EMBO journal, 13(7), pp.1696–1705.PMID: 8157008. 10.1002/j.1460-2075.1994.tb06434.x PMC395002 8157008

[14] Rangel-Garcia CI, Salvador C, Chavez‐Garcia K, Diaz‐Bello B, Lopez‐Gonzalez Z, Vazquez‐Cruz L, Angel Vazquez‐Martinez J, Ortiz‐Navarrete V, Riveros‐Rosas H, Escobar LI. 2021. Identification of a unique endoplasmic retention motif in the Xenopus GIRK5 channel and its contribution to oocyte maturation. FEBS open bio, 11(4), pp.1093–1108.PMID: 33565726. 10.1002/2211-5463.13113 PMC8016131 33565726

[15] Keller SH, Lindstrom J, Ellisman M, Taylor P. 2001. Adjacent basic amino acid residues recognized by the COP I complex and ubiquitination govern endoplasmic reticulum to cell surface trafficking of the nicotinic acetylcholine receptor α-subunit. Journal of Biological Chemistry, 276(21), pp.18384–18391.PMID: 11279119. 10.1074/jbc.M100691200 11279119

[16] Calebiro D, Godbole A (2018). Internalization of G-protein-coupled receptors: implication in receptor function, physiology and diseases. Best Pract Res Clin Endocrinol Metab.

[17] Siddiqui FS, Iqbal Z, Radosevich JA (1992). Changes in the expression of the tumor-associated antigen recognized by monoclonal antibody 44–3A6 in A549 cells due to calcium. Tumor biology.

